# Exogenously Applied Cyclitols and Biosynthesized Silver Nanoparticles Affect the Soluble Carbohydrate Profiles of Wheat (*Triticum aestivum* L.) Seedling

**DOI:** 10.3390/plants12081627

**Published:** 2023-04-12

**Authors:** Lesław B. Lahuta, Joanna Szablińska-Piernik, Karolina Stałanowska, Marcin Horbowicz, Ryszard J. Górecki, Viorica Railean, Paweł Pomastowski, Bogusław Buszewski

**Affiliations:** 1 Department of Plant Physiology, Genetics and Biotechnology, University of Warmia and Mazury, Oczapowskiego Street 1A/103, 10-719 Olsztyn, Poland; 2 Department of Infectious, Invasive Diseases and Veterinary Administration, Institute of Veterinary Medicine, Nicolaus Copernicus University in Torun, Gagarina 7, 87-100 Toruń, Poland; 3 Interdisciplinary Center for Modern Technologies, Nicolaus Copernicus University in Torun, 87-100 Toruń, Poland

**Keywords:** wheat, germination, silver nanoparticles, inositol, pinitol, glutathione, carbohydrates

## Abstract

Cyclitols, such as *myo*-inositol and its isomers and methyl derivatives (i.e., d-*chiro*-inositol and d-pinitol (3-*O*-methyl-*chiro*-inositol)), are classified as osmolytes and osmoprotectants and are significantly involved in plant responses to abiotic stresses, such as drought, salinity and cold. Moreover, d-pinitol demonstrates a synergistic effect with glutathione (GSH), increasing its antioxidant properties. However, the role of cyclitols in plant protection against stresses caused by metal nanoparticles is not yet known. Therefore, the present study examined the effects of *myo*-inositol, d-*chiro*-inositol and d-pinitol on wheat germination, seedling growth and changes in the profile of soluble carbohydrates in response to biologically synthesized silver nanoparticles ((Bio)Ag NPs). It was found that cyclitols were absorbed by germinating grains and transported within the growing seedlings but this process was disrupted by (Bio)Ag NPs. Cyclitols applied alone induced sucrose and 1-kestose accumulation in seedlings slightly, while (Bio)Ag NP doubled the concentrations of both sugars. This coincided with a decrease in monosaccharides; i.e., fructose and glucose. Cyclitols and (Bio)Ag NPs present in the endosperm resulted in reductions in monosaccharides, maltose and maltotriose, with no effect on sucrose and 1-kestose. Similar changes occurred in seedlings developing from primed grains. Cyclitols that accumulated in grain and seedlings during grain priming with d-pinitol and glutathione did not prevent the phytotoxic effects of (Bio)Ag NPs.

## 1. Introduction

Nanomaterials can have both positive and negative effects on plant growth [1,2]. This depends on the properties of the nanomaterials, as well as the species, the stage of plant development and the route of contact between the nanomaterial and the plant; i.e., through seeds, roots or leaves [3,4]. Among the various types of nanomaterials released into the environment, silver nanoparticles (Ag NPs) seem to be among the most important due to their biocidal activity [5,6]. This has led to an in increase in the use of Ag NPs in many industries, such as for textiles, electronics, food storage and processing, healthcare, cosmetics and agronomy [7]. The antibacterial and antifungal properties of Ag NPs make them interesting for use in agriculture to control crop diseases caused by various pathogenic bacteria and fungi [8,9] and against insect pests [10]. Moreover, Ag NPs also show some beneficial effects on plants [11,12,13], especially under stress conditions, such as salinity, heat stress and drought [14,15]. Ag NPs affect the biosynthesis of many phytohormones and signaling pathways in plant cells under stress conditions [15,16] and can increase the activity of antioxidant enzymes [17,18]. It has also been shown that seed priming with nanomaterials seems to be a promising method for increasing plant growth and yield [19,20].

However, both beneficial and harmful effects were found in plants exposed to Ag NPs, which depended on the nanoparticles’ size, their concentration and the coating agents [21,22,23]. The phytotoxic effects of Ag NPs on plants include excessive generation of reactive oxygen species (ROS), which cause lipid peroxidation, protein denaturation and structural modifications to enzymes, as well as damage to membranes, DNA and mitochondria. As a result, cell organelles malfunction and tissue damage occurs, leading to reduced seed germination, seedling development, alternate roots, shoot elongation and leaf numbers [21,22,23,24]. Additionally, the surface oxidation of nanosilver leads to the release of Ag^+^, an ion that is toxic for plants [24]. Thus, the toxicity of silver for plant tissues may depend on both the properties of Ag NPs and the amount of Ag^+^ ions released. This was confirmed by the results of our previous study, which showed that both Ag NPs and Ag^+^ ions inhibit the early growth of seedlings of wheat [25,26] and garden peas [27]. The inhibition of wheat root elongation was accompanied by increased ROS generation and changes in primary metabolism [25,26,28]. However, biologically synthesized silver NPs ((Bio)Ag NPs) show less toxicity for wheat as a result of a reduction in ROS toxicity, presumably due to the antioxidant compounds present in the fruit extract coating the nanoparticles [28]. On the other hand, applying pre-sowing seed treatment with Ag NPs can activate ROS-scavenging enzymes, increasing the range of seed germination and improving the vigor and growth of wheat seedlings [29]. It has also been shown that treating wheat seedlings with a mixture of Ag NPs, nicotinate and KNO_3_ resulted in positive effects on seedlings by regulating pathogen-related protein and ROS scavenging systems [30]. Thus, the types and contents of some of the compounds in seeds, especially those with antioxidant properties, can considerably affect their response to Ag NPs during seed germination.

The major endogenous low-molecular-weight antioxidants in dry and germinated wheat grains are glutathione (γ-l-glutamyl-l-cysteinyl-glycine (GSH)) [31] and ascorbic acid [32]. Both compounds play a vital role in ROS detoxification in an ascorbate-glutathione pathway (called the Asada–Halliwell pathway) under normal and stressful conditions [33], including stress caused by toxic metals [34]. In germinating wheat grains, the ratio of GSH to its oxidation product, which is glutathione disulfide (GSSG), changes in both endosperm and seedlings, affecting the generation and accumulation of H_2_O_2_ [35], and this plays an important signaling role in seedling development [36]. Treatment of wheat seedlings with Ag NPs caused increased expression of mitochondrial superoxide dismutase [37], presumably leading to an overproduction of H_2_O_2_.

An increase in the level of compounds with antioxidant properties in seeds may reduce the phytotoxicity of silver nanoparticles, resulting in increased resistance to Ag NPs during germination and seedling growth. Thus, it would seem to be important to use plant antioxidants (ascorbic acid, tocopherols, GSH, polyphenols, phenolic acids, etc.), as well as other metabolites that exhibit direct (carotenoids, flavonoids [38]) or indirect antioxidative effects, e.g., proline and GABA [39]. Such compounds also include polyhydric cyclic derivatives of monosaccharides (cyclitols), although the mechanism of their antioxidant activity in plants is not fully understood.

The most common cyclitol is *myo*-inositol (MIN), which is found in the cells of all living organisms and is synthesized from glucose [40]. It is used to synthesize phosphatidylinositol, which is an important component of cell membranes and high-energy inositol pyrophosphates [41] and inositol phosphates [42]. In addition, MIN is an essential substrate in the synthesis of phytic acid, which plays an important role in the storage/release of metal ions and phosphate residues in germinating seeds [43]. Moreover, MIN in plants is involved in the synthesis of cell wall compounds [44], galactinol and raffinose family oligosaccharides (RFOs) and ascorbic acid, as well as in plant tolerance to abiotic stresses [45,46].

Recently, MIN has been shown to be involved in the regulation of ROS biosynthesis and antioxidant enzyme activity in plants [47], animals [48] and humans [49]. In addition, it was shown that methyl derivatives of inositol, such as d-pinitol (3-*O*-methyl-d-*chiro*-inositol (PIN)) and l-quebrachitol (2-*O*-methyl-l-inositol), also have free radical scavenging properties [50,51]. Although our recent studies showed that the antioxidant properties of MIN, d-*chiro*-inositol (DCI), PIN and l-quebrachitol are very low compared to flavonoids and GSH, the use of PIN synergistically increased the antioxidant properties of GSH [52]. Thus, it cannot be excluded that some cyclitols may increase the tolerance of germinating seeds to Ag NPs. Cyclitols can also prevent membrane and protein deterioration caused by various abiotic stresses [45,53,54]. The current study evaluated the effects of exogenously applied MIN, DCI and PIN, as well as a mixture of PIN and GSH, on wheat (*Triticum aestivum* L.) grain germination and seedling growth in the presence of (Bio)Ag NPs. The contents and profiles of soluble carbohydrates in seedlings following application of cyclitols and Ag NPs were also analyzed.

## 2. Results

### 2.1. The Uptake of Cyclitols by Wheat Grains during Imbibition—Preliminary Experiment

Imbibition of wheat grains in solutions of *myo*-inositol (MIN), d-*chiro*-inositol (DCI) and d-pinitol (PIN) for 24 h had no effect on grain germination and early seedling growth (Table S1). Both exogenously applied cyclitols (DCI and PIN) were present at higher concentrations in seedlings than in endosperm (Table 1).

Moreover, the application of MIN, PIN and DCI led to an increase in their contents in wheat grains (Table 1). This means that cyclitols were absorbed by grains during imbibition and then were translocated to the growing seedling.

It should be noted that, in seedlings obtained from seeds treated with PIN, its demethylation product, which is DCI, was not found. Similarly, PIN was not found in seedlings obtained from seeds imbibed with DCI solution. Additionally, PIN and DCI uptake did not affect the concentration of MIN in seedlings, while MIN content in endosperm was reduced by 10%. The concentration of total soluble carbohydrates in seedlings from grains imbibed in cyclitols was lower than that in the control. These seedlings contained less sucrose, while the endosperm had lower contents of starch hydrolysis products (maltotriose, maltose and glucose). In addition, the content of 1-kestose was decreased by 10% (Table 1).

### 2.2. The Effect of Cyclitols on Phytotoxicity of Biosynthesized Silver Nanoparticles ((Bio)Ag NPs) to Wheat Seedlings

**Figure 1:**
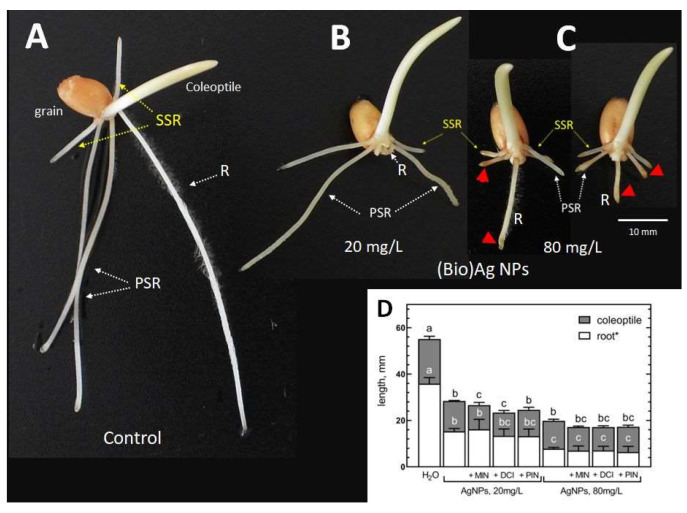
The morphology of 3-day-old seedlings of wheat (*Triticum aestivum* L. cv. Ostka Strzelecka) developing in the absence (**A**) or presence of (Bio)Ag NPs (at (**B**) 20 or (**C**) 80 mg/L). The browning root tips are indicated by red arrows. Abbreviations: R—radicle, PSR—primary seminal roots, SSR—secondary seminal roots. The inhibition of seedling growth (elongation of radicle and coleoptile) by (Bio)Ag NPs alone (at 20 and 80 mg/L) and in mixtures with cyclitols (+MIN, +DCI, +PIN) is shown in (**D**) (values are means (*n* = 3) + SD, and bars with the same letters (a–c) were not significantly (*p* < 0.05) different after ANOVA test and Tukey’s post hoc corrections).

The deleterious effect of (Bio)Ag NPs on the early development of wheat seedlings was manifested by radicle dieback or growth inhibition. As the concentration of nanoparticles increased, inhibition of the elongation of both primary and secondary pairs of seminal roots was also observed (Figure 1).

Moreover, (Bio)Ag NPs at a concentration 80 mg/L caused swelling and browning of the root tips (Figure 1C). The phytotoxicity of (Bio)Ag NPs at both concentrations was not alleviated by cyclitols (Figure 1D, Table 2).

**Figure 2:**
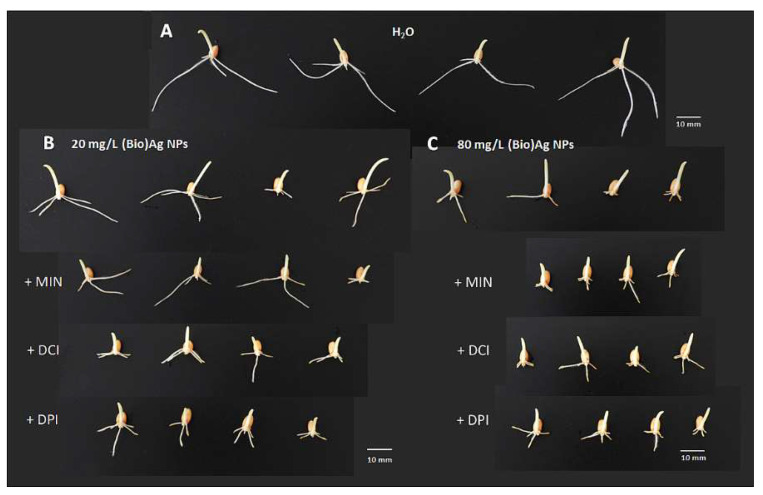
The morphology of 3-day-old seedlings of wheat (*Triticum aestivum* L. cv. Ostka Strzelecka) growing in the absence (**A**) or presence of (Bio)Ag NPs alone (at (**B**) 20 and (**C**) 80 mg/L) and in mixtures with cyclitols—*myo*-inositol (+MIN), d-*chiro*-inositol (+DCI) or d-pinitol (+PIN)—at 100 mM concentration each (**B**,**C**). Horizontal white bars correspond to 10 mm.

Although the damage in wheat seedlings was similar to that caused by (Bio)Ag NPs alone (Figure 2B,C), the addition of cyclitols to the suspension of (Bio)Ag NPs at low concentration (20 mg/L) increased the toxicity of the (Bio)Ag NPs (Figure 2B).

This was manifested by a reduction in seedling growth (Figure 1D) and decreases in FW and water content (Table 2). Such an effect did not occur in seedlings obtained from seeds imbibed in mixtures of cyclitols with (Bio)Ag NPs at 80 mg/L (Figure 2C).

#### Soluble Carbohydrates

**Figure 3:**
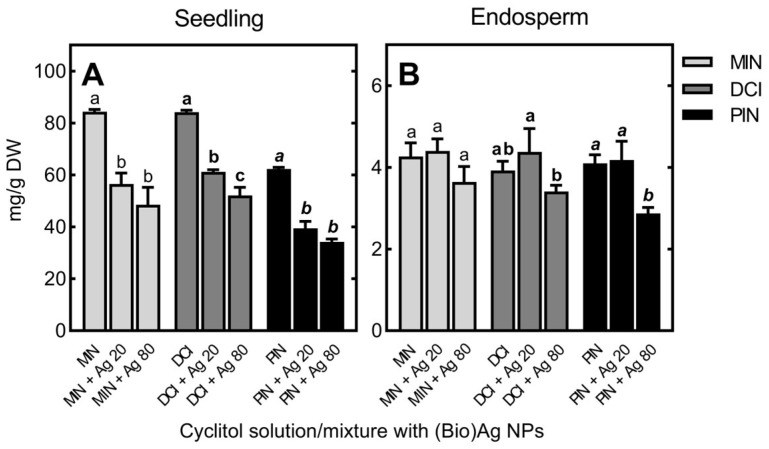
The concentrations of *myo*-inositol (MIN), d-*chiro*-inositol (DCI) and d-pinitol (PIN) in seedlings (**A**) and endosperm (**B**) of wheat (*Triticum aestivum* L. cv. Ostka Strzelecka) germinating for 3 days in solutions of free cyclitols (at 100 mM each) or their mixtures with (Bio)Ag NPs at 20 and 80 mg/L (Ag 20 and Ag 80, respectively). Values are means (*n* = 3) + SD. Bars with the same letters (a–c, **a**–**c**, ***a***–***c***) were not significantly (*p* < 0.05) different after ANOVA test and Tukey’s post hoc corrections.

In control wheat seedlings, endogenous MIN was present at low levels (1.20 mg/g DW), while DCI and PIN were not present at measurable levels. After the 3 day germination process in the presence of MIN, its concentration in wheat seedlings dramatically increased (up to 84.34 mg/g DW, Figure 3A).

For seedlings grown in DCI, the concentration of DCI was as high (84.15 mg/g DW) as that of MIN in seedlings developed in the presence of MIN. However, the uptake of PIN was ca. 25% lower (up to 62.32 mg/g DW; Figure 3A). The content of MIN in the endosperm increased tenfold (from 0.42 to 4.27 mg/g DW), and contents of DCI and PIN were similar (3.92 and 4.10 mg/g DW, respectively; Figure 3B). The uptake of DCI and PIN led to an increase in MIN content in seedlings (from 1.20 to 3.82 and 1.55 mg/g DW, respectively), while there was a slight decrease in its level in endosperm. In addition, low levels of DCI were found in seedlings and endosperm of seeds treated with PIN (0.82 and 0.02 mg/g DW, respectively).

In the seedlings, cyclitol contents decreased when increasing concentrations of (Bio)Ag NPs were used to imbibe wheat seeds (Figure 3A). In the endosperm, decreases in DCI and PIN contents were found only at higher concentrations of (Bio)Ag NPs (80 mg/L; Figure 3B).

**Figure 4:**
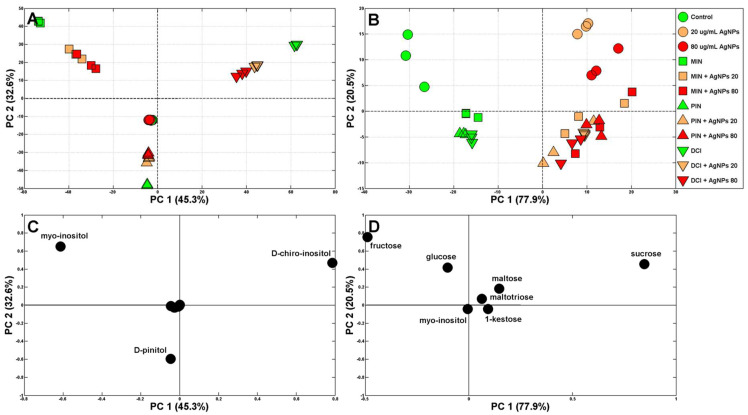
Principal component analysis (PCA) of soluble carbohydrate profiles (with (**A**) and without (**B**) exogenous cyclitols) of wheat seedlings grown in water (control), solutions (green symbols) of *myo*-inositol (MIN), d-pinitol (PIN) and d-*chiro*-inositol (DCI) and mixtures of cyclitols (at 100 mM each) with (Bio)Ag NPs at 20 and 80 mg/L (orange and red symbols, respectively). *Symbols*: circles—control samples and those treated with (Bio)Ag NPs only, squares—samples treated with MIN and MIN + Ag NPs, triangles— samples treated with PIN and PIN + Ag NPs, inverted triangles—samples treated with DCI and DCI + Ag NPs. The PCA loading plots of the soluble carbohydrates are shown in figures (**C**,**D**).

It was to be expected that cyclitols would affect the profiles of soluble carbohydrates in wheat seedlings. Data including MIN, DCI and PIN concentrations were analyzed to determine which had the strongest effect. At that time, the separation of the samples was very clear (Figure 4A), and the main factors differentiating the samples were the cyclitols themselves (Figure 4C), as expected, due to their high concentrations in growing tissues (Figure 3A).

**Figure 5:**
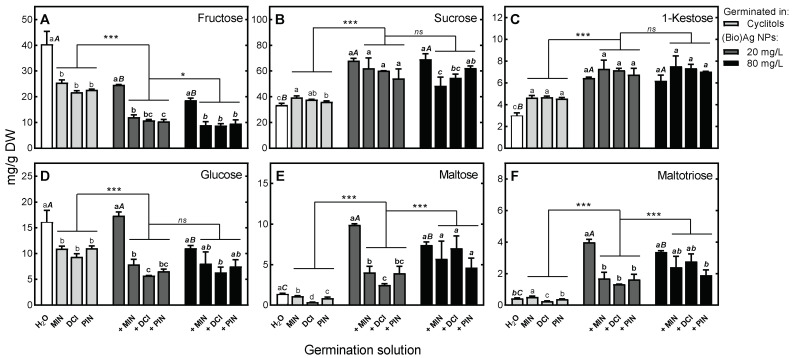
The effects of (Bio)Ag NPs (at 20 and 80 mg/L) and their mixtures with 100 mM *myo*-inositol (MIN), d-*chiro*-inositol (DCI) and d-pinitol (PIN) on soluble carbohydrate contents in 3-day-old wheat (*Triticum aestivum* L. cv. Ostka Strzelecka) seedlings: (**A**) *Fructose*, (**B**) *Sucrose*, (**C**) *1-Kestose*, (**D**) *Glucose*, (**E**) *Maltose*, (**F**) *Maltotriose*. Values are means (*n* = 3) + SD. Bars with the same letters (a–d) were not significantly (*p* < 0.05) different after ANOVA test and Tukey’s post hoc corrections, whereas one to three asterisks (*, ***) denote significant differences (at *p* < 0.05 and 0.001) between effects of cyclitols alone and in mixtures with (Bio)Ag NPs.

Therefore, the data were analyzed again but without these cyclitols (except for the maintained levels of endogenous MIN in control samples and in samples of seedlings treated with (Bio)Ag NPs, DCI and PIN). Control samples and those absorbing exogenous cyclitols in the absence of (Bio)Ag NPs are grouped on the left from PC1 (accounting for 77.9% of the variability), while those treated with (Bio)Ag NPs are on the right (Figure 4B). Moreover, PC2 (accounting for 20.5% of the variability) separated samples treated with (Bio)Ag NPs according to the absence and presence of exogenous cyclitols (in the upper and lower right corners of Figure 4B, respectively). The major sugars influencing samples’ variability were sucrose, fructose and glucose (Figure 4D). They were the quantitatively dominant sugars in control wheat seedlings (16.12, 40.39 and 33.59 mg/g DW, respectively, Figure 5).

In endosperm, maltose, glucose and maltotriose dominated (73.34, 18.99 and 18.95 mg/g DW, respectively). After imbibition of seeds in cyclitol solutions and their accumulation in seedlings (Figure 3A), there were decreases in the contents of fructose, glucose, maltose and maltotriose, while sucrose and 1-kestose increased (Figure 5).

**Figure 6:**
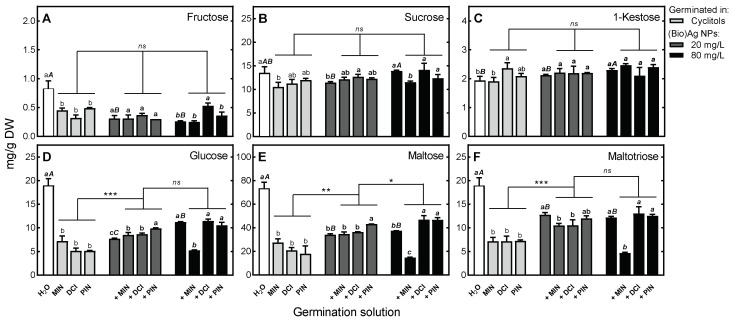
The effect of (Bio)Ag NPs (at 20 and 80 mg/L) and their mixtures with 100 mM *myo*-inositol (MIN), d-*chiro*-inositol (DCI) and d-pinitol (PIN) on the concentrations of soluble carbohydrates in endosperms of 3-day-old wheat (*Triticum aestivum* L. cv. Ostka Strzelecka) seedlings: (**A**) *Fructose*, (**B**) *Sucrose*, (**C**) *1-Kestose*, (**D**) *Glucose*, (**E**) *Maltose*, (**F**) *Maltotriose*. Values are means (*n* = 3) + SD. Bars with the same letters (a) were not significantly (*p* < 0.05) different after ANOVA test and Tukey’s post hoc corrections, whereas one to three asterisks (*, **, ***) denote significant differences (at *p* < 0.05, 0.01 and 0.001) between effects of cyclitols alone and in mixtures with (Bio)Ag NPs.

Application of (Bio)Ag NPs resulted in increased accumulation of sucrose and 1-kestose compared to that caused by cyclitol alone. Although the use of (Bio)Ag NPs caused an increase in maltose and maltotriose contents, the use of mixtures of cyclitol and (Bio)Ag NPs attenuated these processes and caused a decrease in monosaccharide content (Figure 5). On the other hand, in the endosperm, after imbibition of seeds in solutions of cyclitols and (Bio)Ag NPs, the contents of fructose, glucose, maltose and maltotriose decreased (Figure 6).

This effect was not related to the concentration of (Bio)Ag NPs applied or to the presence of DCI or PIN. In contrast, the addition of MIN to (Bio)Ag NPs (at a concentration of 80 mg/L) reduced the concentrations of sucrose, glucose, maltose and maltotriose (Figure 6B,D–F).

### 2.3. The Effect of Grain Priming with d-Pinitol, GSH and Their Mixtures on the Phytotoxicity of (Bio)Ag NPs against Seedlings

**Figure 7:**
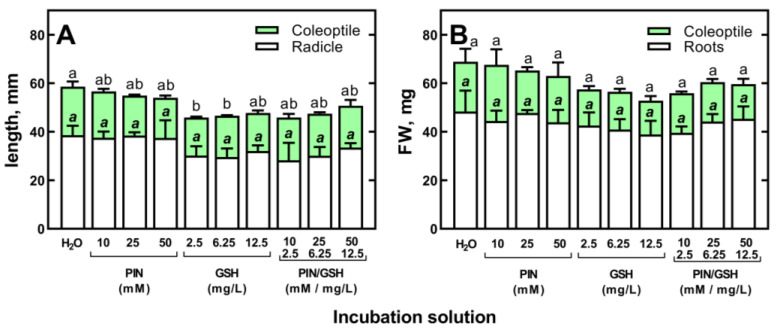
The effect of d-pinitol (PIN; 10, 25 and 50 mM), glutathione (GSH; 2.5, 6.25 and 12.5 μg/mL) and mixtures of PIN/GSH on the length (**A**) and fresh weight (FW) (**B**) of 3-day-old seedlings of wheat (*Triticum aestivum* L. cv. Collada). Control grains were germinated in H_2_O. Values are means of three replicates + SD. Bars with the same letters (a,b, *a*,*b*) were not significantly (*p* < 0.05) different after ANOVA test and Tukey’s post hoc corrections.

For this experiment, grains of the spring wheat cultivar “Collada”, which is characterized by a faster growth rate than cv “Ostka Strzelecka” (data not shown), were used. The germination and development of wheat seedlings from non-primed grains were not affected by PIN at concentrations of 10, 25 and 50 mM or GSH at 2.5, 6.25 and 12.5 mg/L (Figure 7).

**Figure 8:**
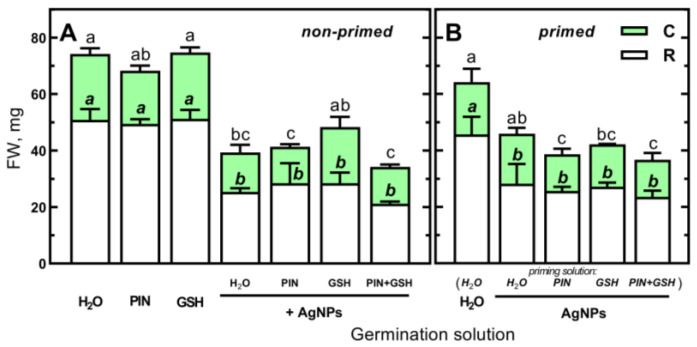
(**A**) The effects of PIN (50 mM), GSH (12.5 mg/L) and (Bio)Ag NPs (at 40 mg/L) alone and in mixtures of PIN, GSH and PIN plus GSH on the fresh weight (FW) of roots (*R*, including scutellum) and coleoptiles (*C*) of 3-day-old seedlings of wheat (*Triticum aestivum* L. cv. Collada). (**B**) The effects of wheat grains priming in H_2_O, PIN, GSH and mixtures of PIN and GSH on the FW of roots and coleoptiles of 3-day-old seedlings of wheat developing in H_2_O or (Bio)Ag NPs (at 40 mg/L). Values are means (*n* = 3) + SD. Bars with the same letters (a–c) were not significantly (*p* < 0.05) different after ANOVA test and Tukey’s post hoc corrections.

Furthermore, the application of mixtures of PIN and GSH had no effect on early seedlings’ growth (Figure 7). Thus, the use of PIN, GSH and their mixture at the highest concentrations examined (50 mM PIN and 12.5 mg/L GSH) to prevent the phytotoxicity of (Bio)Ag NPs is reasonable. However, the addition of PIN, GSH and their mixtures to a suspension of (Bio)Ag NPs did not reduce the toxicity of (Bio)Ag NPs to developing wheat seedlings (Figure 8A).

Furthermore, seedlings grown from wheat grains primed in PIN, GSH and their mixtures were as sensitive as those primed in (Bio)Ag NPs alone (Figure 8B).

#### Soluble Carbohydrates

**Figure 9:**
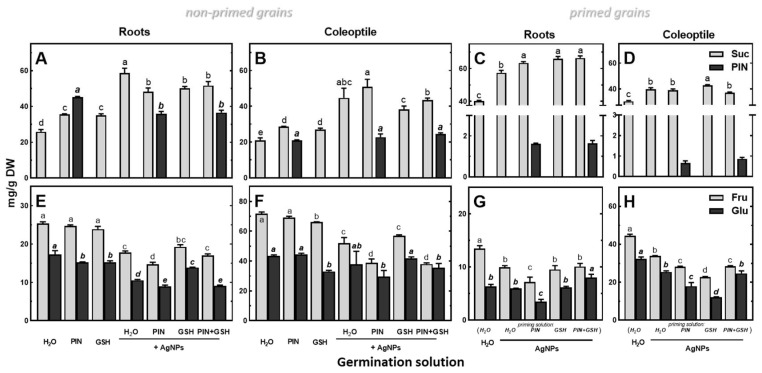
The effects of PIN (50 mM), GSH (12.5 mg/L), (Bio)Ag NPs (at 40 mg/L) and their mixtures on the concentrations of sucrose (*Suc*), d-pinitol (PIN), fructose (*Fru*) and glucose (*Glu*) in roots (**A**,**C**,**E**,**G**) and coleoptiles (**B**,**D**,**F**,**H**) in 3-day-old seedlings of wheat (*Triticum aestivum* L. cv. Collada) obtained from non-primed (**A**,**B**,**E**,**F**) and primed grains (**C**,**D**,**G**,**H**). Values are means (*n* = 3) + SD. Bars with the same letters (a–e) were not significantly (*p* < 0.05) different after ANOVA test and Tukey’s post hoc corrections.

Application of a 50 mM solution of PIN to imbibe seeds resulted in the highest concentration in roots, a lower concentration in coleoptiles and the lowest concentration in the endosperm (45.38, 20.87 and 1.90 mg/g DW, respectively). In response to the application of (Bio)Ag NPs, PIN concentration increased in the endosperm, while it decreased in the roots and remained unchanged in the coleoptiles (Figure 8B and Figure 9A, respectively).

Roots and coleoptiles of wheat seedlings also accumulated sucrose (Figure 9A,B). A similar reaction was revealed in seedlings developing from primed grains (Figure 9C,D). The increase in sucrose content coincided with a decrease in fructose and glucose (Figure 9E–H). Similar changes were also found in the endosperm (Figure S1). Seedlings from non-primed grains developed in PIN and GSH without (Bio)Ag NPs accumulated less sucrose than those in the presence of (Bio)Ag NPs (Figure 9). The addition of PIN and GSH to (Bio)Ag NPs reduced sucrose accumulation in roots (Figure 9A).

In endosperm, the addition of GSH to the suspension of (Bio)Ag NPs was reflected in the maintenance of maltose and maltotriose concentrations at levels as high as in the endosperms of control seedlings and those treated with PIN and GSH only (Figure S1). The opposite effect, a reduction in maltose and maltotriose content in response to (Bio)Ag NPs), was found in the endosperm of seedlings obtained from primed wheat grains (Figure S1).

## 3. Discussion

Silver nanoparticles are phytotoxic to wheat seedlings at an early stage of their development. This manifests as necrosis in the main root, stunting of the seed root, and swelling and browning of the root tips, as reported earlier [25,26,55,56]. Previous reports indicate that damage to the apical zone of wheat roots results from excessive production of ROS in epidermal cells in response to the presence of Ag NPs [25], leading to membrane damage [57], genotoxicity [58] and cell death in root tips [25,55,59]. The degree of damage depends on both the properties of the nanoparticles themselves (size, coating compounds, surface charge) and their concentration [22]. Our current study confirmed that an increase in the concentration of (Bio)Ag NPs from 20 to 40–80 mg/L caused a strong disturbance in the development of the root systems of wheat seedlings and only a slight reduction in the growth of coleoptiles. The reduced growth of the coleoptiles could have resulted from insufficient water uptake by the damaged root system due to the toxicity of nanoparticles and/or silver ions released from them. Although the silver content in wheat tissues was not analyzed in the current study, its uptake and transport in wheat and other plant species have been previously reported [58,60,61] and reviewed [2,62].

It should be also noted that the phytotoxicity of Ag NPs, as well as their biocidal activity (against bacteria, fungi and viruses), can be related to the method of nanoparticle synthesis. Biologically synthesized Ag NPs (using plant extracts or microorganisms) are potentially less toxic than chemically synthesized Ag NPs because they are synthesized using natural biological processes, which generally involve the use of nontoxic or less toxic reagents compared to traditional chemical synthesis methods [63]. Chemical synthesis methods typically require the use of toxic chemicals, such as strong reducing agents and stabilizers, which can remain on the surface of the nanoparticles and affect their biological activity and toxicity [64]. Additionally, the use of biological methods for the synthesis of (Bio)Ag NPs can result in nanoparticles that are more uniform in size and shape, with fewer impurities or defects on their surfaces, which can further reduce their toxicity. Moreover, the functional groups from biological compounds that are used to stabilize the nanoparticles can provide a protective layer that prevents aggregation, reduces the release of silver ions and limits their interaction with biological molecules, which can also reduce their toxicity. Although both chemically and biologically synthesized Ag NPs show antimicrobial activity against Gram-negative and Gram-positive bacteria, (Bio)Ag NPs seem to be more effective, presumably due to their smaller size, greater stability and unique surface chemistry [65]. The presence of organic stabilizing agents on the surface of (Bio)Ag NPs can enhance their interactions with bacterial cells and increase their effectiveness [66]. Moreover, the presence of multiple active compounds in the biological extracts used for synthesis of (Bio)Ag NPs may lead to synergistic effects, enhancing their antimicrobial activity [67].

### 3.1. The Role of Cyclitols in Reducing the Phytotoxic Effects of (Bio)Ag NPs

Our attempt to use cyclitols to protect wheat seedlings against the harmful properties of silver nanoparticles required determining (i) the ability of wheat seeds to absorb these cyclitols through dry grains and/or growing seedlings, (ii) the movement (transport) of cyclitols within seedling tissues and (iii) the metabolism of exogenously applied cyclitols (PIN and DCI). The obtained results confirmed both the uptake of all cyclitols examined here by wheat grains and seedlings and their transport to the growing roots and coleoptiles (Table 1; Figure 3 and Figure 9A–D). This confirms our previous studies on the uptake of exogenous PIN and DCI by developing and maturing seeds of cereals [68] and legumes [69,70]. Furthermore, the higher content of DCI and PIN in the roots of wheat seedlings compared to coleoptiles shown in this study (Figure 9A–D) is consistent with previous data. This also indicates that cyclitol transport between plant organs through both the xylem and phloem occurs [71,72]. In addition, the higher concentrations of cyclitols in the tissues of 3-day-old wheat seedlings growing in their solutions (Figure 3) compared to the cyclitol content in seedlings obtained from grains imbibed with cyclitols for 8 or 24 h confirmed that cyclitols are also taken up by the roots (Figure 9A–D and Table 1, respectively). Interestingly, PIN concentrations in such seedlings were lower than DCI and MIN concentrations (Figure 3). This could mean that the process of cyclitol uptake depends not only on the time of seed and seedling exposure but also on the presence and substrate specificity of cyclitol transporters in root epidermal cells. In *Arabidopsis thaliana*, two of four identified *myo*-inositol transporters were localized in the plasma membrane and one in the tonoplast [73]. The two plasma-localized H^+^/inositol symporters indicate an ability to transport, in addition to MIN, its epimers; i.e., DCI and *scyllo*-inositol [74]. Moreover, one of them can also transport PIN but at a much lower rate [74]. Thus, if the inositol transporters in wheat have similar properties as those in *A. thaliana*, the lower concentration of PIN compared to MIN and DCI (Figure 3) seems to be explainable. Importantly, the drastic reduction in the uptake of all cyclitols investigated in the presence of (Bio)Ag NPs (Figure 3A and Figure 9A) suggests that silver nanoparticles may disrupt membrane inositol transporters.

The metabolism of PIN and DCI in germinating seeds and seedlings is poorly understood. In germinating legume seeds, PIN and DCI concentrations increase due to the hydrolysis of their α-d-galactosyl derivatives accumulated in maturing seeds [75]. However, in these species, PIN and DCI, as well as other cyclitols, such as d-bornesitol, d-ononitol and l-*chiro*-inositol, are also synthesized de novo in vegetative tissues. The metabolic pathway of PIN synthesis from MIN has been explored [76] and reviewed [77], but the pathway of DCI synthesis remains to be explained. It seems that this process occurs through PIN demethylation or MIN epimerization. In our study, only trace amounts of DCI were found in seedlings formed from grains imbibed or germinated in PIN solution. Moreover, there was no increase in MIN content in seedlings treated with DCI or PIN (Table 1). MIN, DCI and PIN were present in wheat seedlings at concentrations as high as those of monosaccharides or sucrose (Figure 3 and Figure 5). This may indicate their important role [78,79] in balancing water homeostasis in the tissues of growing seedlings.

Our preliminary hypothesis that the addition of cyclitols to a (Bio)Ag NP suspension used for seed imbibition could protect wheat seedlings against the toxicity of silver nanoparticles was not confirmed (Figure 1D and Figure 2B,C). The morphological damage in roots was similar to that observed in our previous studies [25,26]. This damage was probably caused by stress and excessive generation of ROS, which led to the death of epidermal cells in root tips. In addition, cyclitols (at 100 mM) added to the (Bio)Ag NP (20 mg/L) suspension even increased the damaging effects of the nanoparticles (Figure 2B, Table 2), possibly by increasing the uptake of silver ions by roots.

### 3.2. GSH and GSH/PIN in The Protection of Wheat Seedlings against (Bio)Ag NPs

Endogenous GSH plays a crucial role in mitochondrial metabolic activity [80] and thiol redox status during seed germination [81]. GSH is also responsible for the maintenance of redox homeostasis in plastids and cytoplasm [82,83]. So far, the possible involvement of GSH, which contains a cysteine moiety in its structure, in chelating Ag^+^ ions released from Ag NPs has not been described [84]. Free cysteine is such a compound, and it completely inhibited the effect of Ag NPs, probably by chelating the Ag^+^ ions then formed [85]. In our study, the application of (Bio)Ag NPs and GSH at low concentrations (ca. 2.5 and 10-fold, respectively) was not effective in decreasing the deleterious effects of Ag NPs (Figure 8). Furthermore, addition of PIN at a concentration of 50 mM to GSH solution (based on previous results of in vitro analyses [52]) also had no effect on seedlings’ growth. This was presumably due to low concentrations of GSH or its reaction with (Bio)Ag NPs.

### 3.3. The Effect of (Bio)Ag NPs and Cyclitols on Soluble Carbohydrates in Seedlings

In tissues of wheat seedlings after 3 (experiments two and three) or 4 (experiment one) days of germination, the major sugars were monosaccharides (fructose and glucose) and sucrose, which accounted for ca. 94% of TSCs, while maltose, maltotriose and glucose quantitatively dominated in the endosperm (accounting for 90% of TSCs). These differences confirm the mobilization of starch as the primary energy reserve in the starchy endosperm [85]. In contrast, in the scutellum, there is a conversion of starch hydrolysis products (maltose, glucose) into sucrose [86] and its further transport to the roots and coleoptile, where it is hydrolyzed into fructose and glucose [87]. The activities of different isoforms of α-amylase change in a spatiotemporal manner [88].

The decreases in the concentrations of glucose, maltose and maltotriose in the endosperm with (Bio)Ag NPs treatment (Figure 6) may have been due to the inhibition of the activity of the amylases. It has been shown that inhibition of α-amylase by the allosteric inhibitor acarbose in germinating wheat grains leads to the accumulation of soluble α-gluco-oligosaccharides without affecting the first stage of germination [89]. In our current study, there was no effect of (Bio) Ag NPs on the germination of wheat grains. The opposite, stimulatory effect of Ag NPs on the activity of α-amylase in germinating rice grains has previously been demonstrated [90]. Our current study showed a decrease in the rate of starch hydrolysis in endosperm under the influence of both cyclitols and (Bio)Ag NPs (at both concentrations, 20 and 80 mg/L). This process was further dramatically accelerated by the presence of MIN (Figure 6D–F), indicating that this cyclitol can alter starch metabolism. Up to now, the possible binding of cyclitols to the allosteric site of α-amylase (as has been found in the case of some flavonoids [91,92]) or the replacement of Ca^2+^ ions from the active site of amylase [91] by Ag^+^/Ag NPs, affecting enzyme activity, have not been revealed.

The increasing concentration of MIN in seedlings may also interfere with hormonal homeostasis during grain germination [93] due to possible inhibition of the hydrolysis of IAA conjugated with inositol. Decreased levels of active IAA can, in turn, interfere with other hormones, such as ethylene, abscisic acid and gibberellins, which play a crucial role in starch mobilization and growth in wheat seedlings [94]. Moreover, coleoptile and root elongation may also be influenced by changes in the ratio of sucrose to MIN, which regulates cell elongation, via the vacuole-localized inositol transporter, which facilitates the import of MIN from the vacuole into the cytoplasm [95].

(Bio)Ag NPs in wheat seedlings stimulated both the accumulation of starch degradation end products and sucrose and 1-kestose (Figure 5 and Figure 9A–D). This may have been a result of the inability of growth-restricted seedlings to metabolize sugars released from or over-accumulated in the scutellum [86]. It was found that the cyclitols present there reduced the accumulation of maltose and maltotriose (Figure 5E,F) and favored a decrease in monosaccharide content (Figure 5A,D), which may suggest the substitution of sugars as energy and carbon sources for growing tissues. The utilization of MIN (and/or other cyclitols) for the synthesis of precursors for cell wall polysaccharides [42,96] also seems to be important [87] due to the rapid elongation of the roots and coleoptile.

## 4. Materials and Methods

### 4.1. Preparation of (Bio)Ag NP Suspensions

In the present study, the silver nanocomposites employed were previously synthesized and characterized using a complementary approach [97]. The silver nanocomposites synthesized with the biological method (in post-culture medium from the *Lactobacillus curvatus* MEVP1 strain) were found to have a complex structure consisting of a metallic silver core and organic branching coats. The transmission electron microscopy (TEM) analyses revealed that the mean size of the synthesized nanoparticles was 16.5 ± 5.91 nm (and the size ranged between 5 and 30 nm), and selected area electron diffraction (SAED) patterns proved the crystalline nature of the synthesized nanoparticles. The registered hydrodynamic diameter was around 400 nm, whereas the silver metal core was <50 nm; the nanocomposites were stable even after 7 days, showing a zeta potential value equal to or higher than −30 mV. Moreover, the TEM results showed that the nanoparticles were homogenously dispersed on the organic matrix [97].

The stock suspension of (Bio)Ag NPs (200 mg/L) was prepared on the day of use by adding 23.6 mg of dry Ag NPs to 118 mL of double-distilled water (DDW) and applying sonication for 45 min (Sonic-3, 310 W, 40 KHz, POLSONIC Pałczyński, Poland). Then, the stock suspension was diluted with DDW to obtain (Bio)Ag NPs at concentrations of 20, 40 and 80 mg/L. The cyclitols *myo*-inositol (MIN), d-*chiro*-inositol (DCI) and d-pinitol (PIN) (Sigma-Aldrich, Saint Louis, MO, USA) were dissolved in DDW to obtain a concentration of 100 mM for each cyclitol. Mixtures of (Bio)Ag NPs at concentrations of 20 and 80 mg/L with cyclitol (at 100 mM) were prepared by adding appropriate amounts of cyclitols to (Bio)Ag NPs suspensions. Mixtures were vortexed for 2 min and sonicated for 45 min immediately before being used for experiments.

### 4.2. The Uptake and Transport of Cyclitols in Germinating Wheat Grains—A Preliminary Study

Wheat grains of the “Ostka Strzelecka” cultivar (purchased from Hodowla Roślin Strzelce, Płock city, Poland) were used for the present study. Grains were imbibed in 100 mM solutions of MIN, DCI or PIN for 24 h at 20 °C in the dark. Then, the grains (30 for each of the three replicates) were washed with DDW and germinated in paper towels (wetted with DDW) for 3 days at 20 °C in the darkness (ILP 115 growth chamber, Pol-Eko, Poland). The germinability (G, %) and percentage of developing seedlings (DS, %) were calculated as described previously [25]. After measurement of the length of the coleoptile and the longest root, the whole seedlings (roots with coleoptile and scutellum) and endosperms were weighed, frozen in liquid nitrogen and stored in an ultra-freezer (at −76 °C) for 2 days. Next, tissues were freeze-dried for 48 h (shelf freeze-dryer, Alpha 1–2 LD, Martin Christ, Osterode am Harz, Göttingen, Germany). The fresh weight (FW) and dry weight (DW) were expressed in mg per seedling and endosperm. The water content (WC) was calculated as the difference between the FW and DW and expressed as a percentage of the FW. The freeze-dried and pulverized tissues were taken for soluble carbohydrate analyses.

### 4.3. The Effect of Exogenous Cyclitols on the Phytotoxicity of (Bio)AgNPs

Wheat grains (cv ”Ostka Strzelecka”) were germinated in Petri dishes (120 × 20 mm) in 15 mL of DDW, (Bio)Ag NPs suspensions with concentrations of 20 and 80 mg/L, 100 mM MIN, 100 mM DCI, 100 mM PIN and mixtures of (Bio)Ag NPs (20 and 80 mg/L) and each cyclitol (at 100 mM concentration) for 3 days at 20 °C in the dark. Each treatment was undertaken in three replicates on separate Petri dishes containing 40 grains each. After measurement of the length of seedlings, as well as the FW and DW, the freeze-dried and pulverized whole seedlings (coleoptile with scutellum and roots) and endosperms were taken for soluble carbohydrate analyses.

### 4.4. The Effects of Seed Priming with d-Pinitol, GSH and Their Mixtures on The Phytotoxicity of (Bio)AgNPs

Grains of the spring wheat cultivar “Collada”, which is characterized by a faster growth rate than cv “Ostka Strzelecka”, were germinated in Petri dishes separately in DDW (control), PIN (50 mM), GSH (12.5 mg/L), (Bio)Ag NPs (40 mg/L) and mixtures of (Bio)Ag NPs and PIN, GSH and PIN plus GSH for 3 days. The concentrations of d-pinitol and GSH used here were chosen based on the results of our previous experiments, where they led to synergistic effects between d-pinitol and GSH relative to the antioxidant potential [52], as well as a preliminary test of the effects of both compounds on wheat grain germination/seedling growth.

The second portion of grains was primed as follows: grains were imbibed separately in 50 mM PIN, 12.5 mg/L GSH or their mixtures for 4 h and then dried under laboratory conditions (at 22 °C and 35% air relative humidity (RH)) for 3 days. Grains were then placed into Petri dishes containing a solution of (Bio)Ag NPs at 40 mg/L and germinated for 3 days. The 3-day-old seedlings obtained from non-primed and primed seeds were separated on the coleoptile, roots and endosperm for analyses of their FW, DW, water content (WC) and soluble carbohydrates profile.

### 4.5. Soluble Carbohydrate Analyses

The soluble carbohydrates were analyzed using a high-resolution gas chromatography method described previously [93]. Briefly, sugars were extracted from 40–42 mg of dry pulverized tissues with 50% ethanol containing xylitol as the internal standard. After heating (at 90 °C for 30 min), the homogenate was centrifuged (20,000× *g* for 20 min at 4 °C) and aliquots of clear supernatant were filtered using micro-spin filters (PVDF, 0.2 μm, Thermo Fisher Scientific, Loughborough, UK). A part of the filtrate was evaporated to dryness in a speed vacuum rotary evaporator (JW Electronic, Warsaw, Poland). Carbohydrates were derivatized with a mixture of trimethylsilyl imidazole and pyridine (1:1, *v*/*v*), and TMS derivatives of soluble carbohydrates were analyzed in a gas chromatograph (GC 2010, Shimadzu, Japan) equipped with a Zebron ZB-1 capillary column (15 m length, 0.25 mm diameter, 0.1 μm film, Phenomenex, Torrance, CA, USA) and flame-ionization detector, at conditions described earlier [98]. Carbohydrates were quantified by using original standards of glucose, fructose, galactose, sucrose, maltose, maltotriose, 1-kestose, MIN, DCI and PIN (purchased from Sigma-Aldrich, Saint Louis, MO, USA).

## 5. Conclusions

The results of the study revealed a similar metabolic response in the germinating wheat seeds/seedlings to silver nanoparticles and exogenously applied natural sugar derivatives (cyclitols). Regardless of whether the active factor led to the inhibition of seedling growth (as in the case of silver nanoparticles) or not (as in the case of cyclitols), the tissues of the growing organs accumulated sucrose and 1-kestose, while in the storage tissues (endosperm), the contents of starch degradation end products—glucose, maltose and maltotriose—decreased. This means that both (Bio)Ag NPs and cyclitols affect starch mobilization and sugar metabolism. Moreover, the (Bio)Ag NP-dependent inhibition of the uptake of exogenous cyclitols by grains/seedlings is a new observation, valuable for a deeper explanation of the mechanisms of silver phytotoxicity. Additionally, application of cyclitols alone and in a mixture with glutathione is not effective in the prevention of silver nanoparticles’ toxicity to wheat.

## Figures and Tables

**Table 1:** The soluble carbohydrate contents in 4-day-old wheat (*Triticum aestivum* L. cv. Ostka Strzelecka) seedlings after 24 h seed imbibition in double-distilled water (DDW) or aqueous solutions of *myo*-inositol (MIN), d-*chiro*-inositol (DCI) or d-pinitol (PIN) at a concentration of 100 mM each. Values (in mg/g DW) are means of three replicates. The same letters by the values indicate that there were no significant (*p* < 0.05) differences after ANOVA test and Tukey’s post hoc corrections.

	Solution	Carbohydrate									
		Fructose	Glucose	Sucrose	Maltose	Maltotriose	Kestose	MIN	DCI	PIN	Total
Seedling	DDW	33.63 a	22.90 a	39.54 a	5.64 a	1.37 a	2.05 a	0.97 b	-	-	106.10 a
	MIN	33.21 a	21.99 a	32.21 b	3.82 a	1.37 a	2.20 a	1.31 a	-	-	96.10 b
	DCI	34.04 a	21.09 a	31.77 b	5.76 a	2.05 a	2.08 a	0.99 b	2.29	-	97.17 b
	PIN	32.56 a	22.55 a	29.97 b	5.01 a	1.49 a	1.74 a	0.92 b	-	2.92	100.07 b
Endosperm	DDW	0.56 a	18.38 a	11.97 a	88.44 a	19.87 a	1.21 a	0.55 a	-	-	140.97 a
	MIN	0.69 a	20.96 a	13.32 a	85.57 a	22.37 a	1.04 ab	0.61 a	-	-	144.55 a
	DCI	0.44 a	18.15 ab	10.39 a	84.23 a	21.81 a	0.91 b	0.49 a	0.05	-	109.30 b
	PIN	0.43 a	14.82 b	11.84 a	64.56 b	15.84 b	0.95 b	0.49 a	-	0.27	136.47 a

**Table 2:** The germinability (G), fresh weight (FW), dry weight (DW) and water content (WC) in seedlings of wheat (*Triticum aestivum* L. cv. Ostka Strzelecka) developing for 3 days in double-distilled water (DDW), (Bio)Ag NPs (at 20 and 80 mg/L), cyclitols (each at 100 mM) and mixtures of (Bio)Ag NPs and cyclitols (data for cyclitols are means from treatment with MIN, DCI and PIN). Values are means of three replicates. The same letters by the values indicate that there were no significant (*p* < 0.005) differences after ANOVA test and Tukey’s post hoc corrections.

	Germination Solution					
	H_2_O	Cyclitols *	(Bio)Ag NPs			
			20 mg/L	80 mg/L	20 mg/L + Cyclitols *	80 mg/L + Cyclitols *
G, %	95.83 b	95.56 b	95.00 b	98.33 a	92.22 b	91.39 b
FW, mg	56.10 a	45.11 a	37.09 a	25.07 b	29.19 b	26.49 b
DW, mg	5.18 a	4.74 ab	3.94 c	3.58 c	5.02 a	4.51 bc
WC, %	90.76 a	89.43 a	89.26 a	85.59 b	82.61 c	82.62 c

*, mean for treatment with MIN, DCI and PIN.

## Data Availability

Not applicable.

## Supplementary Materials

The following supporting information can be downloaded at: https://www.mdpi.com/article/10.3390/plants12081627/s1. Table S1. The germinability (G), the percentage of developing seedlings (DS), the length of the coleoptile and longest seminal root and the fresh and dry weight (FW and DW, respectively) of wheat seedlings (*Triticum aestivum* L. cv. Ostka Strzelecka). These seedlings were developed for 3 days (in double-distilled water (DDW)) from grains imbibed for 24 h in DDW or water solutions of *myo*-inositol (MIN), d-*chiro*-inositol (DCI) or d-pinitol (PIN) at concentrations of 100 mM each. Values are means of three replicates. The same superscript letters by the values indicate no significant (*p* < 0.05) differences after ANOVA test and Tukey’s post hoc corrections. Figure S1. The effects of d-pinitol (PIN, 50 mM), glutathione (GSH, 12.5 mg/L), (Bio)Ag NPs (at 40 mg/L) and their mixtures on the concentrations of sucrose, glucose (A,B), maltose and maltotriose (C,D) in the endosperm of 3 day old seedlings of wheat (*Triticum aestivum* L. cv. Collada) developed from non-primed (A,C) and primed grains (B,D). Values are means (*n* = 3) + SD. Bars with the same letters (a–e) were not significantly (*p* < 0.05) different after ANOVA test and Tukey’s post hoc corrections.
