# Supplementary material for: Exogenously Applied Cyclitols and Biosynthesized Silver Nanoparticles Affect the Soluble Carbohydrate Profiles of Wheat (Triticum aestivum L.) Seedling
Source: Plants (Basel). 2023 Apr 12;12(8):1627. doi: 10.3390/plants12081627 (PMC10145852; doi:10.3390/plants12081627)
Supplement: Supplementary file 1 [file plants-12-01627-s001.zip › plants-2283905-supplementary.pdf]

Supplementary Material

Table S1. The germinability (G), percent of developing seedlings (DS) and the length of the coleoptile and longest seminal root, and fresh and dry weight (FW and DW, respectively) of wheat seedlings (*Triticum aestivum* L. cv. Ostka Strzelecka). These seedlings were developed for 3 days (in double distilled water, DDW) from grains imbibed for 24 hours in DDW or water solutions of *myo*-inositol (MIN), D-*chiro*-inositol (DCI) or D-pinitol (PIN), at concentrations of 100 mM each. Values are means of three replicates. The same superscript letters by the values indicate no significant (P < 0.05) differences after ANOVA test and Tukey's post hoc corrections.

Imbibition	G (%)	DS (%)	Length, mm		FW, mg		DW, mg	
			Root*	Coleoptile	Seedling**	Endosperm	Seedling**	Endosperm
DDW	92.2 ^{a}	72.22 ^{a}	43.2 ^{a}	22.1 ^{a}	87.06 ^{a}	66.65 ^{a}	8.30 ^{a}	33.97 ^{a}
MIN	87.8 ^{a}	81.11 ^{a}	48.6 ^{a}	22.3 ^{a}	80.90 ^{a}	60.05 ^{a}	7.39 ^{a}	31.59 ^{a}
DCI	86.7 ^{a}	77.78 ^{a}	56.5 ^{a}	23.3 ^{a}	86.43 ^{a}	59.97 ^{a}	8.33 ^{a}	31.77 ^{a}
PIN	93.3 ^{a}	81.11 ^{a}	52.7 ^{a}	27.2 ^{a}	95.31 ^{a}	58.66 ^{a}	8.58 ^{a}	30.05 ^{a}

* - The length of radicle; **- a total of 3 seminal roots, coleoptile and scutellum

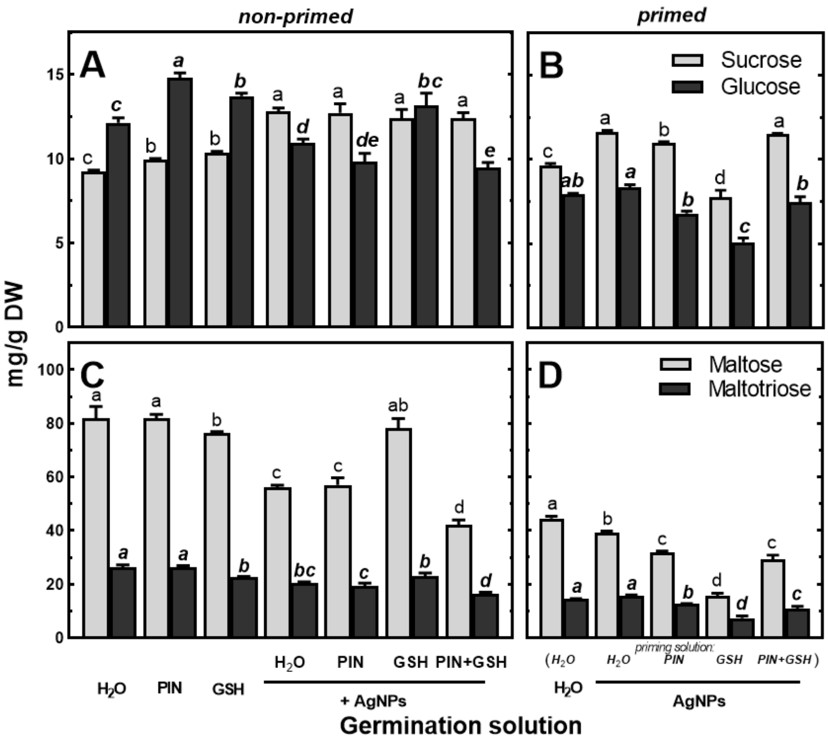

Figure S1. The effect of D-pinitol (PIN, 50 mM), glutathione (GSH, 12.5 mg/L), (Bio)Ag NPs (at 40 mg/L) and their mixtures on the concentrations of sucrose, glucose (**A**, **B**), maltose and maltotriose (**C**, **D**) in the endosperm of 3-day-old seedlings of wheat (*Triticum aestivum* L. cv. Collada) developed from non-primed (**A**, **C**) and primed grains (**B**, **D**). Values are means (n=3) + SD. Bars with the same letters (a-e) are not significantly (P < 0.05) different after ANOVA test and Tukey's post hoc corrections.
